# Anti-*Helicobacter pylori* Properties of the Ant-Venom Peptide Bicarinalin

**DOI:** 10.3390/toxins10010021

**Published:** 2017-12-29

**Authors:** Jesus Guzman, Nathan Téné, Axel Touchard, Denis Castillo, Haouaria Belkhelfa, Laila Haddioui-Hbabi, Michel Treilhou, Michel Sauvain

**Affiliations:** 1 Laboratorios de Investigación y Desarrollo, Universidad Peruana Cayetano Heredia (UPCH), Lima 34, Peru; guzman_j29@hotmail.com (J.G.); denis.castillo.p@upch.pe (D.C.); michel.sauvain@ird.fr (M.S.); 2 EA7417-BTSB, Université Fédérale Toulouse Midi-Pyrénées, INU Champollion, 81012 Albi, France; nathan.tene@univ-jfc.fr (N.T.); T.Axel@hotmail.fr (A.T.); 3 Fonderephar, Université de Toulouse, Faculté des Sciences Pharmaceutiques, 31062 Toulouse, France; haouaria_belkhelfa@hotmail.com (H.B.); laila.haddioui@fonderephar.com (L.H.-H.); 4 UMR 152 PHARMADEV, Université de Toulouse, IRD, 31062 Toulouse, France

**Keywords:** bicarinalin, antimicrobial peptide, *Helicobacter pylori*, gastric cells, bacterial adhesion, SEM

## Abstract

The venom peptide bicarinalin, previously isolated from the ant *Tetramorium bicarinatum*, is an antimicrobial agent with a broad spectrum of activity. In this study, we investigate the potential of bicarinalin as a novel agent against *Helicobacter pylori*, which causes several gastric diseases. First, the effects of synthetic bicarinalin have been tested against *Helicobacter pylori*: one ATCC strain, and forty-four isolated from stomach ulcer biopsies of Peruvian patients. Then the cytoxicity of bicarinalin on human gastric cells and murine peritoneal macrophages was measured using XTT and MTT assays, respectively. Finally, the preventive effect of bicarinalin was evaluated by scanning electron microscopy using an adherence assay of *H. pylori* on human gastric cells treated with bicarinalin. This peptide has a potent antibacterial activity at the same magnitude as four antibiotics currently used in therapies against *H. pylori*. Bicarinalin also inhibited adherence of *H. pylori* to gastric cells with an IC_50_ of 0.12 μg·mL^−1^ and had low toxicity for human cells. Scanning electron microscopy confirmed that bicarinalin can significantly decrease the density of *H. pylori* on gastric cells. We conclude that Bicarinalin is a promising compound for the development of a novel and effective anti-*H. pylori* agent for both curative and preventive use.

## 1. Introduction

*Helicobacter pylori* is a unique bacteria able to colonize human stomach mucosa [1,2]. This helix-shaped Gram-negative bacteria expresses outer membrane proteins which enable it to bind epithelial gastric cells, and secretes ureases which enable it to overcome stomach acidity. It is estimated that half of the world’s population is infected with *H. pylori*, making this pathogen one of the most common bacterial infections globally [3,4]. The colonization of stomachs by *H. pylori* results in gastric inflammation (gastritis), and a persistent colonization is recognized as the leading factor in the development of gastric ulcers and cancers [5,6]. *H. pylori* can be eradicated by a proton pump inhibitor combined with two or three antibiotics (i.e., amoxicillin, clarithromycin, metronidazole, and levofloxacin) [7]. However, in recent years, the overuse of this therapeutic strategy has promoted the emergence of antibiotic resistant strains, and antibiotic resistance is now the main reason for treatment failure. Therefore, finding alternative anti-*H. pylori* therapies is of considerable interest [6,8]. Several natural products have already been proven to actively suppress *H. pylori*, contributing significantly to the therapeutic arsenal against gastrointestinal infections and diseases [9].

In this context, antimicrobial peptides (AMPs) may provide an alternative approach in the treatment of *H. pylori*. These peptides are naturally found in a variety of organisms and are an essential part of the innate immune system of both invertebrates and vertebrates [10,11,12]. AMPs can generally be defined as short (10 to 60 amino acids) with an overall positive charge (generally +2 to +9). They have a substantial proportion of hydrophobic residues (>30%), enabling them to disrupt bacterial membranes. They therefore could be used against a broad range of bacteria including some that are resistant to conventional antibiotics [13]. Consequently, they have great potential as new antibiotics against both human and animal pathogens, although, to date, clinical trials have mostly demonstrated their efficacy as topical agents [14].

Peptides are the predominant class of toxins in most arthropod venoms, and multiple AMPs have been reported in the venoms of scorpions [15], spiders [16], centipedes [17], wasps [18] and ants [19,20]. Our research group has previously isolated the antimicrobial polycationic and c-terminally amidated peptide bicarinalin in the venom of the myrmicine ant *Tetramorium bicarinatum*. Recent antimicrobial bioassay-based studies on several pathogens confirmed that bicarinalin is an effective and fast-acting molecule with a broad spectrum of antimicrobial activity and a moderate cytotoxicity against human lymphocytes [13,21]. Several studies argued that AMPs, including bicarinalin, are suitable for the development of novel preservatives in the food industry [22]. Given this, were the peptide used as a preservative it might also prevent some gastric diseases by acting against *H. pylori* once ingested.

There have been no studies to date of venom peptides as potential anti-*H. pylori* agents. Consequently, we embarked on an investigation of bicarinalin with a view towards the development of an antimicrobial agent to protect the human stomach against the colonization of *H. pylori*. In this study, we demonstrate that *H. pylori* strains isolated from Peruvian patients present antimicrobial resistance to the antibiotics clarithromycin and levofloxacin and sensitivity to metronidazole. Then, we show that bicarinalin peptide has a strong antimicrobial activity against both reference and Peruvian-patient strains of *H. pylori*. Finally, we show that bicarinalin has low cytotoxicity for both peritoneal macrophages and gastric cells, but efficiently limited the adherence of *H. pylori* to human gastric cells. 

## 2. Results

Clarithromycin, levofloxacin, metronidazole and amoxicillin are the conventional drugs used in a triple therapy to treat stomach infection by the gram-negative bacteria *H. pylori* even though the eradication rate is currently less than 80% in most parts of the world. Antibiotic resistance is the main reason for treatment failure. In this study, we isolated 44 clinical strains of *H. pylori* from cultures of gastric tissues of 95 biopsies obtained from Peruvian patients with dyspeptic symptoms. In Table 1, we show the antimicrobial activities of conventional antibiotics against both clinical strains and the reference ATCC strain. The results show that conventional antibiotics are more efficient with clinical strains except for metronidazole, which has a MIC_50_ 16-fold higher against ATCC strain.

**Figure 1:**
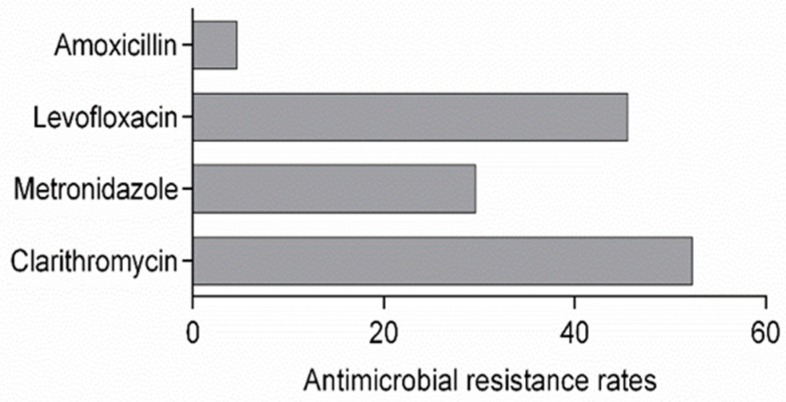
Antimicrobial resistance rates of Peruvian clinical *H. pylori* strains to conventional antibiotics according the EUCAST antimicrobial breakpoints for *H. pylori*: clarithromycin: S ≤ 0.7 μmol·L^−1^, R > 1.4 μmol·L^−1^; metronidazole: S ≤ 46.8 μmol·L^−1^, R > 46.8 μmol·L^−1^; levofloxacin: S ≤ 1.34 μmol·L^−1^, R > 1.34 μmol·L^−1^; amoxicillin: S ≤ 0.28 μmol·L^−1^, R > 0.28 μmol·L^−1^.

A previous study conducted in Peru in 2008 highlighted that the antimicrobial resistance rates of *H. pylori* fluctuated between 6.7% and 27% for clarithromycin, was around 50% for metronidazole, 36.9% for levofloxacin, and 7% for amoxicillin [23]. In our study, the resistance rates to clarithromycin and levofloxacin in Peru increased to 52.3% and 45.5%, respectively (Figure 1). In contrast, the resistance rate to metronidazole decreased to 29.6%, while the resistance rate to amoxicillin was stable at 4.6%.

Cationic peptides have great potential in the development of novel antimicrobial agents, particularly for topical application. They are in general strongly antimicrobial and are efficient against a broad spectrum of pathogens, including those resistant to conventional antibiotics. The bicarinalin peptide displayed antimicrobial activity against all *H. pylori* and was 3.3 times more potent against clinical strains than the ATCC strain (Table 1). 

**Figure 2:**
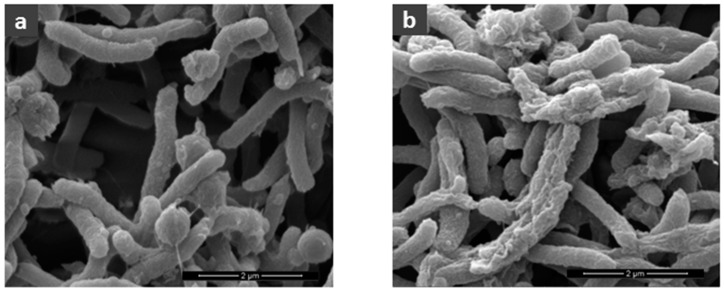
Scanning-electron microscopy analysis of *H. pilori*. (**a**) Without antimicrobial peptide; (**b**) with bicarinalin (60 μg·mL^−1^). (SEM mag = 20,000).

We conducted a scanning electron microscopy analysis to directly evaluate the effect of bicarinalin against *Helicobacter pylori*. As shown in Figure 2, the microscopy revealed the membrane perturbation of *H. pylori* bacteria treated with 60 μg·mL^−1^ of bicarinalin, although membrane perturbation appeared slight even with 10 μg·mL^−1^. The ability of antimicrobial peptides to effect membrane permeability is well known [24], including for bicarinalin [13].

**Figure 3:**
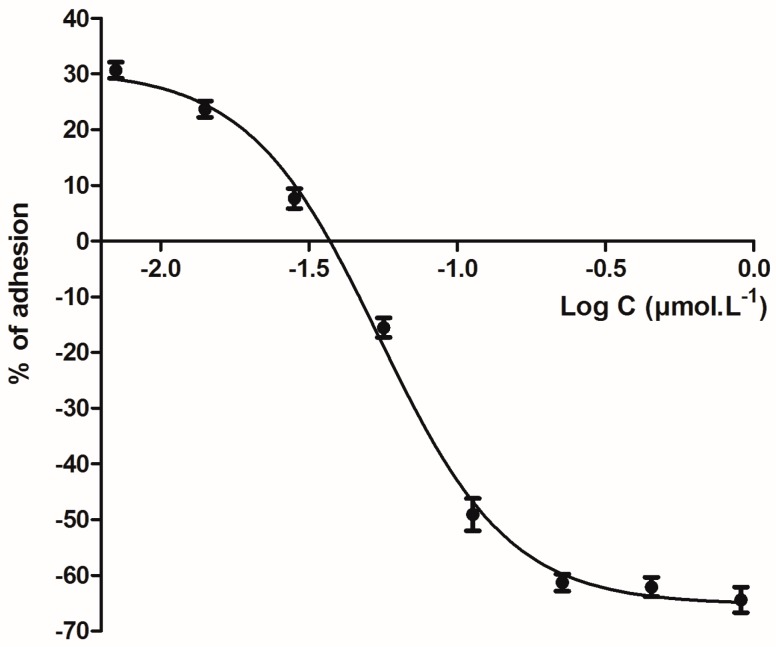
Anti-adhesive effect of *helicobacter pylori* on gastric cells.

The second aim of this study was to evaluate the effect of bicarinalin on the inhibition of adhesion of *H. pylori* (ATCC strain) on the gastric cell line (N87). The adhesion of *Helicobacter pylori* to gastric cells in the presence of bicarinalin was measured by radioactivity. We established that 50% of the adhesion (IC_50_) is inhibited by a concentration of 0.12 μg·mL^−1^ (Log IC_50_ = −0.92) i.e., 0.054 μmol·L^−1^. Above 0.56 μg·mL^−1^ (Log −0.25) i.e., 0.25 μmol·L^−1^ of bicarinalin, the inhibition of *H. pylori* adhesion on gastric cells reaches its maximum. On the other hand, at concentrations lower than 0.032 μg·mL^−1^ (log −1.5), bicarinalin no longer exhibits any significant anti-adhesive effect (Figure 3).

**Figure 4:**
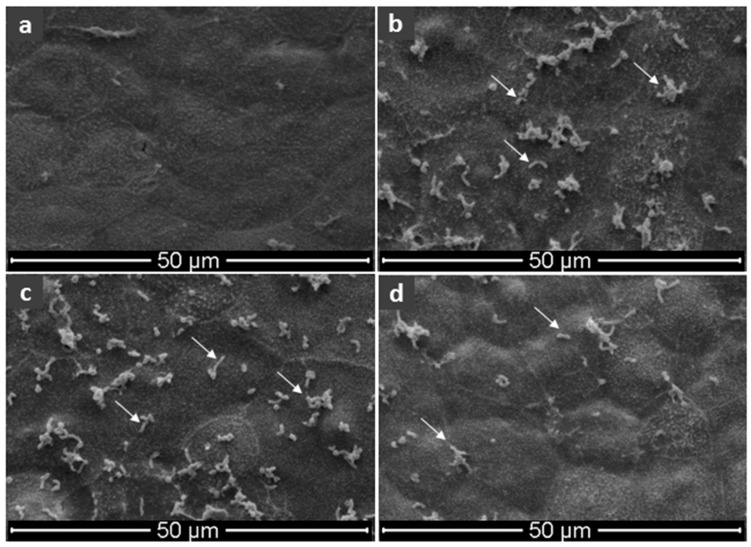
SEM images of cultured cellular carpet of human stomach: (**a**) no *H. pylori*; (**b**) *H. pylori* present; (**c**) *H. pylori* present with 0.015 μg·mL^−1^ of bicarinalin or (**d**) with 0.25 μg·mL^−1^ of bicarinalin (SEM mag = 1000; Arrows show single or aggregated bacteria).

On the basis of these results, electron microscopy images were performed in vitro on gastric cells (N87); with no *H. pylori*, with *H. pylori* but without bicarinalin treatment, and with *H. pylori* with bicarinalin treatment. With no *H. pylori*, Figure 4a shows a uniform cell carpet, while with *H. pylori* and without bicarinalin, isolated and aggregated bacteria adhering to the gastric cell carpet are observed (Figure 4b). The effect of bicarinalin on the adhesion of *H. pylori* to gastric cells was investigated at 0.015 and 0.25 μg·mL^−1^ (Figure 4c,d respectively). The similarity between Figure 4b,c does not reveal an effect of bicarinalin at 0.015 μg·mL^−1^. In contrast, Figure 4d shows a significant decrease in the density of bacteria on the surface of the cellular carpet. This result is in accordance with those obtained by the radioactive counting of Figure 3, which shows that 0.25 μg·mL^−1^ provides the maximum inhibition of adhesion. However, electron microscopy shows that the maximum inhibition observed by radioactivity does not mean that no bacteria adhere, since some bacteria and aggregates are still present.

To complete this study, the cytotoxicity of bicarinalin on both peritoneal macrophages and gastric cell lines was determined. Cytotoxic concentrations of 50% were measured at 39.2 μmol·L^−1^ and 1.7 μmol·L^−1^, respectively (Table 2). This resulted in a selectivity index (SI) of 39 between macrophages and *H. pylori*, and 17 between gastric cells and *H. pylori*. The selectivity index was determined as the ratio of the concentration of the bicarinalin that reduced *Helicobacter pylori* viability to 50% (MIC_50_) to the concentration of the bicarinalin needed to inhibit the cytopathic effect to 50% of the control cells (CC_50_ of gastric cells and peritoneal macrophages).

## 3. Discussion

Antimicrobial peptides (AMPs) have promise as antibacterial agents to overcome multi-drug resistant bacteria, however, systemic therapies have yet to be launched. Currently, topical application of AMPs is preferred. However, this raises the question of whether AMPs could be used to treat human pathogens colonizing mucosal surfaces, such as *Helicobacter pylori*. Previous studies have highlighted the remarkable antimicrobial activity of the ant venom peptide bicarinalin on a broad range of human pathogens and have suggested that it could be developed as a food preservative. Continuing with this idea, we investigated the effect of bicarinalin on the stomach bacteria, *H. pylori*.

Bicarinalin has a direct cytotoxic effect on *H. pylori* (ATCC 43504 strain), having a MIC_50_ of 3.9 μmol·L^−1^ that is comparable to that of anti-*H. pylori* peptides isolated from the frog *Odorrana grahami* (Odorranaina, MIC_50_ of 8.1 μmol·L^−1^) [25], those isolated from the fishes *Epinephelus coioides* and *Pardachirus marmoratus*, Epi-1 (MIC_50_ = 8.1 μmol·L^−1^) and pardaxin (>7.5 μmol·L^−1^) [26]. Nevertheless, bicarinalin was also active against forty-four clinical strains of *H. pylori* and requires a lower molar concentration to inhibit the growth of 50% of clinical isolates (MIC_50_ = 0.99 μmol·L^−1^), suggesting a better activity profile than clarithromycin, levofloxacin and metronidazole (Table 1). The cytotoxicity of bicarinalin on the gastric cells is quite similar to the MIC_50_ for the ATCC strain, which would indicate that bicarinalin is not ideal for use as a curative treatment. However, the cytotoxicity of bicarinalin on the gastric cell line NCI-N87 (IC_50_ > 1.7 μmol·L^−1^) compared to the MIC_50_ for clinical strains, led to a selectivity index higher than 17 (Table 2) which could makes it an interesting lead molecule to overcome *H. pylori* even though extending works should be carried out to try to decrease the cytotoxicity on gastric cells.

Anti-adhesion therapy is an attractive novel approach to fight drug-resistant bacteria [27]. This approach has been validated by several studies, which include *H. pylori* [28,29,30]. Bicarinalin inhibits the adhesion of *H. pylori* to the gastric cell model with an IC_50_ < 0.098 μmol·L^−1^ (<0.25 μg·mL^−1^), which is about forty times lower than the MIC_50_ obtained in the antimicrobial assay of the ATCC *H. pylori* strain (43504) and around ten times lower than the bicarinalin MIC_50_ tested on the *H. pylori* strains isolated from patients. These results suggest that bicarinalin can inhibit a key step in the establishment of infection of the gastric epithelial cells by *H. pylori* in addition to the direct cytotoxic effect observed at higher concentrations.

Electron microscopy confirms a significant reduction in the adhesion of bacteria to gastric cells from 0.25 μg·mL^−1^ of bicarinalin, whereas visible effects on the plasma membrane of bacteria do not appear until 10 μg·mL^−1^. This suggests that the integrity of the bacterial membrane and its ability to adhere to gastric cells is impacted at lower concentrations than those needed to observe membrane perturbations by SEM. Therefore bicarinalin can be considered effective against *H. pylori* at relatively low concentrations: less than 1 μg·mL^−1^ with an SI always greater than 10.

## 4. Conclusions

In summary, our data show that bicarinalin has important direct antimicrobial action on different strains of *H. pylori* isolated from dyspeptic patients as well as the reference strain. Furthermore, bicarinalin has an indirect action on *H. pylori* by inhibiting bacterial adhesion on the surface of gastric epithelial cells. Therefore, we conclude that bicarinalin could be considered as a novel alternative compound for curative and preventive therapies against *H. pylori* and contribute to controlling this emerging global health problem and the issues associated with antimicrobial resistance. However, future investigations should be conducted to study the activity of bicarinalin as well as its stability in vivo.

## 5. Materials and Methods

### 5.1. Bicarinalin Synthesis

Bicarinalin is a C-terminally amidated peptide of twenty residues (KIKIPWGKVKDFLVGGMKAV-NH_2_) that was synthesized on a Liberty microwave assisted automated peptide synthesizer (CEM, Saclay, France) at a higher than 99% purity grade, as previously described in Rifflet et al. [21]. The purity and the molecular identity of the synthetic peptide were controlled using MALDI-TOF mass spectrometry.

### 5.2. Microorganism Strains and Growth Conditions

The *H. pylori* clinical strains were obtained from patients recruited at the Gastroenterology Service of the Cayetano Heredia Medical Clinic in Peru who presented symptoms of dyspepsia. Gastric tissues from dyspeptic patients were extracted via endoscopic gastric biopsy and were transported in 1 mL of BHI broth/FBS/glycerol (*v*/*v*/*v*; 80/10/10) at 4 °C. Gastric tissues were homogenized using a 40 μm diameter cell disintegration mesh incorporated into a BDFalcon^®^ tube. The resulting homogenate was subjected to serial dilutions of 10^−1^ and 10^−2^; and cultivated on blood agar plates composed of a BHI agar supplemented with: 10% *v*/*v* defibrinated sheep blood/water, Amphotericin B, and Skirrow Campylobacter selective supplement. The plates were incubated at 37 °C in an atmosphere of 5% O_2_ and 10% of CO_2_ for five to seven days [31]. Small transparent colonies were grown and were then re-cultured on fresh blood agar plates. The isolated *H. pylori* strains were characterized by microbiological screening according to culture characteristics (small, slightly hemolytic), morphological features (curved bacillary or spiral Gram negative bacteria), biochemical tests (catalase, oxidase and positive urease), and conventional PCR (23S rRNA gene) [32,33].The characterized *H. pylori* strains were collected and resuspended in 5 mL of BHI/FBS/glycerol (*v*/*v*/*v*; 80/10/10). The suspensions were homogenized by vortexing and stored at −70 °C.

Brain heart infusion (BHI) broth, fetal bovine serum (FBS) amphotericin B were supplied by Sigma Aldrich France. Glycerol was supplied by HiMedia USA. Defibrinated sheep blood and Campylobacter selective supplement containing Vancomycin, Trimetropin and Polymyxin B was supplied by OXOID France. The *H. pylori* reference strain (ATCC 43504) used was purchased from the ATCC^®^. The BHI broth, FBS and the reference antibiotics; clarithromycin, metronidazole, amoxicillin and levofloxacin were supplied by Sigma Aldrich USA. IsoVitalex was supplied by BD BBL USA.

### 5.3. Antimicrobial Assays

Minimal inhibitory concentrations (MIC) of the four reference antibiotics plus Bicarinalin were determined by a standard broth microdilution assay following the guidelines of the Clinical and Laboratory Standards Institute (CLSI) [34]. Bacterial innocula of *H. pylori* from both clinical strains and the reference strain (ATCC 43504) were suspended at 10^7^ to 10^8^ CFU·mL^−1^ in BHI broth medium/FBS/IsoVitalex (*v*/*v*/*v*; 89/10/1) [35,36]. In addition, we evaluated the activity of four antimicrobials used in eradication therapy of *H. pylori*: clarithromycin, metronidazole, amoxicillin and levofloxacin. These were added to the medium at different concentrations: from 0.25 μg·mL^−1^ to 2 μg·mL^−1^, from 2 μg·mL^−1^ to 16 μg·mL^−1^, from 0.03 μg·mL^−1^ to 0.24 μg·mL^−1^ and from 0.25 μg·mL^−1^ to 2 μg·mL^−1^. The serial dilutions for each antibiotic were calculated based on the cut-off points recommended by the European Committee on Antimicrobial Susceptibility Testing (EUCAST) [37]. The synthetic peptide bicarinalin was added to the medium at several concentrations between 0.1 and 10 μg·mL^−1^. The cultures were incubated at 37 °C in an atmosphere of 5% O_2_ and 10% CO_2_ for 72 h. The MIC values were visually determined and were defined as the lowest concentration where antibiotics or bicarinalin induced a complete inhibition of visible growth in the culture. The MIC of both antibiotics and bicarinalin were calculated by a Probit logistic regression analysis of percentages of inhibition accumulated versus the distribution of MICs observed in the isolated strains of *H. pylori* for each antibiotic and bicarinalin. Strains were categorized as sensitive or resistant according to cut-off points recommended by EUCAST [37]. The MIC assays were performed in triplicate.

### 5.4. Anti-Adherence Effect

The NCI-N87 gastric cell line was cultured in RPMI 1640 medium supplemented with 10% FBS, 1% IsoVitalex, 1% penicillin and streptomycin at 37 °C in a 5% CO_2_ humidified atmosphere. After 48 h of incubation, the single-cell layer obtained was removed with 5 mL of 0.05% trypsin-EDTA solution for 5 min at 37 °C. Trypsin was inactivated by the addition of 10 mL of RPMI 1640 medium supplemented with 10% FBS. The cells were harvested by centrifugation at 3500 *g* for 5 min at 20 °C [38]. The cell viability was checked by trypan blue assay and the cell suspension was adjusted to 1 × 10^6^ viable cells·mL^−1^. 

*H. pylori* strain ATCC 43504 cultures (2 × 10^8^ bacteria·mL^−1^) were inoculated in 10 mL BHI broth with 30 μL of tritium adenine solution (1 μCi) and incubated for 48 h at 37 °C in an atmosphere of 5% O_2_ and 10% CO_2_. To eliminate non-incorporated cells in the cultures, the bacteria were washed three time using PBS buffer (centrifugation 2500 *g*/10 min at 5 °C). The inhibition of adhesion was evaluated on a 96-well plate previously prepared by placing 500 μL/well of 2 × 10^8^ bacteria·mL^−1^ suspension of treated bacteria and the gastric cells (N87). The cells were treated with serial dilutions of bicarinalin concentrations between 0.9 and 0.007 μmol·L^−1^ (2 and 0.015 μg·mL^−1^) at 37 °C for 24 h. Then, non-adherent bacteria were eliminated by PBS washing (three times). At the end of the treatment, the gastric cells received 500 μL of a lysis solution (SDS 0.1% (*w*/*v* in NaOH 5 mol·L^−1^) and were then incubated at 37 °C for 12 h [39]. Radioactivity was measured with a beta-liquid scintillation system (Perkin Elmer, San Diego, CA, USA). The percentages of inhibition of adherence was calculated as follows:

$$\%\;\mathrm{Adherence}\;\mathrm{inhibition}=\frac{\left(\mathrm{CPM}\;\mathrm{control}-\mathrm{CPM}\;\mathrm{treatment}\right)\times 100}{\mathrm{CPM}\;\mathrm{control}}\;[\mathrm{CPM}=\mathrm{counts}\;\mathrm{per}\;\mathrm{minute}]$$

The required concentration of bicarinalin to inhibit the adherence of 50% of bacteria was expressed as IC_50_, which was calculated by a logistic regression analysis of probit.

### 5.5. Cytotoxicity of Bicarinalin

The cytotoxic effect of bicarinalin on human gastric cells (NCI-N87) and murine peritoneal macrophages (RAW 264) were determined with XTT and MTT assays, respectively [40].

Suspensions of trypsinized gastric cells (10^6^ cells·mL^−1^) were incubated with phenol at 0.5% in the medium (as a positive control) or serial dilutions of bicarinalin concentrations between 0.9 and 0.007 μmol·L^−1^ (2 and 0.015 μg·mL^−1^) for 24 h at 37 °C. Then, 50 μL of XTT was added to each well and incubated at 37 °C for 2 h. Absorbance was read at 450 nm in the Chameleon-Hidex^®^ plate reader.

Murine macrophages were cultured in RPMI 1640 medium and incubated at 37 °C in a 5% CO_2_ atmosphere. Then, 0.05% of a trypsin-EDTA solution (Invitrogen^®^) was added and incubated for 2 min. Subsequently, 100 μL of suspensions of the macrophages (1 × 10^5^ macrophages·mL^−1^) were distributed in each well and incubated for 24 h. Then, the microdilution assay was prepared in a system of three serial dilutions of 50 μmol·L^−1^ maximum concentration of bicarinalin and incubated for 48 h at 37 °C in 5% CO_2_. MTT reagent was added for 4 h and the reaction was stopped by adding 100 μL of a solution of isopropanol/SDS/water (*v*/*v*/*v*; 50/10/40) over 30 min. Finally, absorbance was read at 570 nm in the Chameleon-Hidex^®^ plate reader.

All experiments were conducted in triplicate. The CC_50_ values for both gastric cells and macrophages were obtained by a logistic regression analysis Probit based on the calculation of the percentage of viability calculated as follows:

$$\%\;\mathrm{Viability}=\frac{\mathrm{Abs}\;\mathrm{control}-\mathrm{Abs}\;\mathrm{treatment}}{\mathrm{Abs}\;\mathrm{control}}\times 100$$

### 5.6. Scanning Electron Microscopy

#### 5.6.1. Helicobacter SEM

*Helicobacter pylori* ATCC 43504 cultured on blood agar was used to prepare five tubes of inoculum of 1 × 10^8^ CFU·mL^−1^ using brucella broth. Then, bicarinalin was added to each tube to achieve final concentrations of 15, 30, 60, 120, and 240 μg·mL^−1^ which were incubated for 1 h. The bacteria were washed three times using PBS buffer and centrifuged at 3000 rpm during 10 min at 5 °C. The PBS was replaced by 2% glutaraldehyde in 0.1 mol·L^−1^ Sorensen phosphate buffer (pH 7.4). 

#### 5.6.2. Gastric cells SEM

*H. pylori* ATCC 43504 cultured on blood agar was used to prepare an initial inoculum of 1 × 10^7^ to 1 × 10^8^ CFU·mL^−1^. After washing the inoculum, the optical density was adjusted at 2 × 10^8^ CFU·mL^−1^ using the cell culture medium RPMI 1640. This last inoculum was then added to a microwell plate with previously adhered gastric cells, and two concentrations of bicarinalin (0.25 and 0.016 μg·mL^−1^). After two hours of incubation under microaerophilic conditions, cell culture medium was removed and replaced by 2% glutaraldehyde in 0.1 mol·L^−1^ Sorensen’s phosphate buffer (pH 7.4).

#### 5.6.3. Scanning Electron Microscopy

The bacterial cells (alone or with gastric cells) were fixed in 2% glutaraldehyde in 0.1 mol·L^−1^ Sorensen phosphate buffer (pH 7.4) for at least 4 h at 4 °C. After sedimentation, the pellets were resuspended in water and adhered to poly-lysine coated glass coverslips. The bacteria were then dehydrated in a graded ethanol series and dried by critical point drying with a Leica EM CPD 300. The samples were coated with 6 nm platinum on a Leica EM Med 020 before being examined on a FEI Quanta 250 FEG scanning electron microscope, at an accelerating voltage of 5 kV.

## Figures and Tables

**Table 1:** Antibacterial activities of bicarinalin and reference antibiotics against *H. pylori* strains.

*H. pylori* Strain	MIC_50_ μmol·L^−1^ (μg·mL^−1^)				
	Bicarinalin	Clarithromycin	Levofloxacin	Metronidazole	Amoxicillin
ATCC 43504	3.9 (8.6)	0.042 (0.03)	0.17 (0.06)	374.4 (64)	0.035 (0.014)
Peruvian patients	0.99 (2.2)	0.66 (0.5)	1.94 (0.7)	23.4 (4)	<0.082 (0.03)

**Table 2:** Cytotoxicity of bicarinalin.

	CC_50_ (μmol·L^−1^)	SI
Peritoneal macrophages (Balb/C)	39.2	>39 ^a^
Gastric cells (N87)	1.7 *	>17 ^b^
a: SI = CC_50_ */MIC_50_; b: SI = CC_50_ **/IC_50_		
